# Epidemiological and Clinical Profile of Dermatoses Observed in Chronic Hemodialysis Patients at the National Teaching Hospital (NTH-HKM) of Cotonou, Benin

**DOI:** 10.1155/2020/9186309

**Published:** 2020-02-13

**Authors:** Hugues Adégbidi, Fabrice Akpadjan, Odile Houngbo, Jacques Vigan, Bérénice Dégboé, Nadège Agbessi, Christiane Koudoukpo, Félix Atadokpèdé

**Affiliations:** ^1^Department of Dermatology and Venereology of the National Teaching Hospital, Hubert Koutoukou Maga (NTH-HKM) of Cotonou, Cotonou, Benin; ^2^Faculty of Health Sciences, University of Abomey-Calavi, Cotonou, Benin; ^3^Nephrology and Hemodialysis Department of the NTH-HKM of Cotonou, Cotonou, Benin; ^4^Department of Dermatology and Venereology of the Departmental and Teaching Hospital of Borgou-Alibori, Parakou, Benin; ^5^Faculty of Medicine, University of Parakou, Parakou, Benin

## Abstract

*Introduction*. Dermatological damage in chronic hemodialysis patients is not uncommon. In Benin, to date, no study on the dermatological manifestations of chronic hemodialysis patients has been carried out. However, the presence of cutaneous signs is evident in these patients, and the need for dermatological care is not negligible. The objective of this study was to identify the epidemiological and clinical profile of the main dermatological manifestations presented by chronic hemodialysis patients at the NTH-HKM of Cotonou (Benin).

**Methods:**

This was a descriptive cross-sectional study conducted in chronic hemodialysis patients from May 15th to September 15th, 2018. Included were all patients seen during the study period who had been on hemodialysis for at least three months, had at least one dermatological manifestation, and gave verbal or written consent. Chronic hemodialysis patients who did not wish to participate in the survey were excluded.

**Results:**

87 patients were included in the study for a hospital frequency of 33.8%. The sex ratio (male to female) was 2. The median age was 49 years (IQ [40.75–59]). Median age in hemodialysis was 36 months with two weekly sessions. The main dermatological manifestations were xerosis (48.3%), pruritus (34.5%), alopecia (14%), nail dystrophy (9.2%), equisegmented nails (8%), and melanoderma (8%). Pruritus was associated with a longer duration of hemodialysis sessions (*p*=0.01), while xerosis, alopecia, and melanoderma were associated with seniority in hemodialysis.

**Conclusion:**

Cutaneous manifestations in hemodialysis patients were frequent and dominated by xerosis, pruritus, and alopecia. Factors associated with some of these dermatologic manifestations were seniority in hemodialysis, long duration of the hemodialysis session, and female gender.

## 1. Introduction

The kidneys are the “chief chemists of the body.” When chronic end-stage renal disease occurs, it becomes necessary to use renal replacement therapies, including hemodialysis [1].

Hemodialysis therefore makes it possible to prolong the life of end-stage chronic renal failure at the cost of various side effects [2]. This may be due to one of the following factors: chronic renal failure itself, concomitant medication, an adverse effect of the dialysis technique used, comorbidities, or the combination of several of these factors [3].

Among these side effects, the cutaneous signs could be emblematic. The skin, this external organ, “mirror of the organism,” an obvious actor in social relations and communication, reveals these damages which are displaying, even disabling, a source of discomfort and significant psychological impact [3–5].

It is estimated that one in two patients with chronic renal failure and dialysis has at least one dermatological complaint. Pruritus, xerosis, and pigment disorders are the most common skin signs found in these patients [3, 6].

This study is justified by the fact that no study on this subject has been carried out in Benin to date. However, the incidence of hemodialysis is increasing. According to statistics, not yet published by the Nephrology Department of the NTH-HKM in Cotonou, 48 new cases were recorded in 2015, 91 in 2016, and 162 in 2017. The presence of skin signs is evident, and the request for dermatological care for these patients is significant. The aim of this study was to find out the epidemiological and clinical profile of dermatological manifestations in chronic hemodialysis patients at the NTH-HKM of Cotonou. This will make it possible to raise awareness among potential candidates for hemodialysis and, if possible, prevent the occurrence of these skin abnormalities.

## 2. Patients and Methods

This was a descriptive cross-sectional study that took place at the dialysis unit of the nephrology department at the NTH-HKM of Cotonou, Benin. It lasted 4 months (from May 15th to September 15th, 2018). The target population was hemodialysis patients who had been on hemodialysis for at least three months. All these patients were interviewed and systematically examined (completely undressed) by a dermatologist before the dialysis session. Patients with at least one dermatosis were included in the study. Definitively included were all patients seen during the study period who had been on hemodialysis for at least three months, had at least one dermatological manifestation, and had given their verbal or written consent. The main dermatological manifestations sought were keratosis pilaris, early canitis, subcutaneous nodules, eczema, scabiosis, acne, melanonychia, digital hippocratism, mycosis, melanoderma, equisegmented nails, nail dystrophy, alopecia, pruritus, and cutaneous xerosis. Chronic hemodialysis patients who did not wish to participate in the survey were excluded. The agreement of the management of the NTH-HKM and the Ethics Committee of the Faculty of Health Sciences had also been obtained before the study began. Additional information was obtained in the “Hemodialysis Booklet,” which also contained the biological examinations. Hemodialysis sessions were scheduled twice a week for each patient at the NTH-HKM of Cotonou. The membrane was made of polysulfone, and the dialysate flow rate was 500 ml/min. Using a survey form, sociodemographic, clinical, and biological data were collected and analysed in the SPSS version 20 software; Pearson's Chi-square and associated tests as appropriate were used. The probability values *p* < 0.05 were retained as statistically significant.

## 3. Results

Epidemiologically, 257 hemodialysis patients were identified in this survey. Among them, 87 had presented at least one dermatological manifestation, representing a hospital frequency of 33.8%. Some presented only functional signs (pruritus) and others presented cutaneous physical signs associated or not with functional signs. The sex ratio was 2, with a median age of 49 years (extremes 24–75 years), and the age group over 50 years was the most represented (48.8%). The median duration of hemodialysis was 36 months (range 3–252 months). Half of the patients spent 5 hours per hemodialysis session. (Table 1).

**Figure 1:**
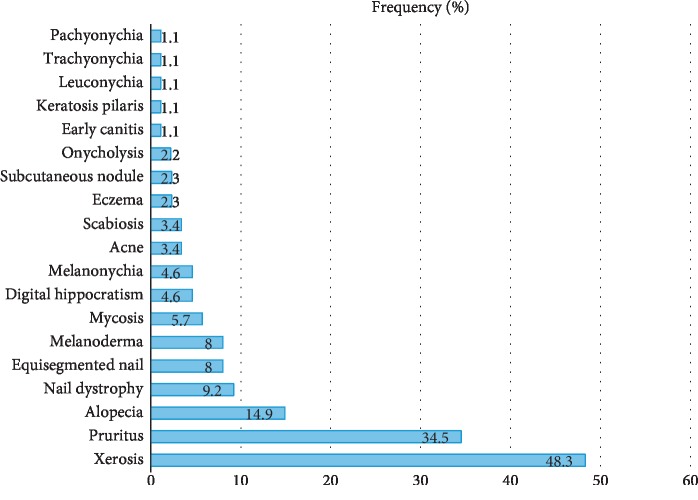
Main dermatological manifestations observed in chronic hemodialysis patients at the NTH-HKM of Cotonou (Benin).

Clinically, 74 patients (85%) had at least one physical skin sign with or without associated functional signs. Pruritus was the main functional sign observed in 34.5% of patients. Skin manifestations were dominated by xerosis (48.3% with an ichthyosiform appearance in 12.6%). Phanerial damage was represented by nail damage (22.7%) and alopecia (14%) (Figure 1).

Regarding associations (Tables 2–5), the longer the duration of hemodialysis sessions, the greater the risk of patients developing pruritus (*p*=0.01). Xerosis (*p*=0.01), alopecia (*p*=0.03), and melanoderma (*p*=0.04) were related to seniority in hemodialysis. Women on hemodialysis had a higher risk of developing alopecia (*p*=0.004) .

## 4. Discussion

**Figure 2:**
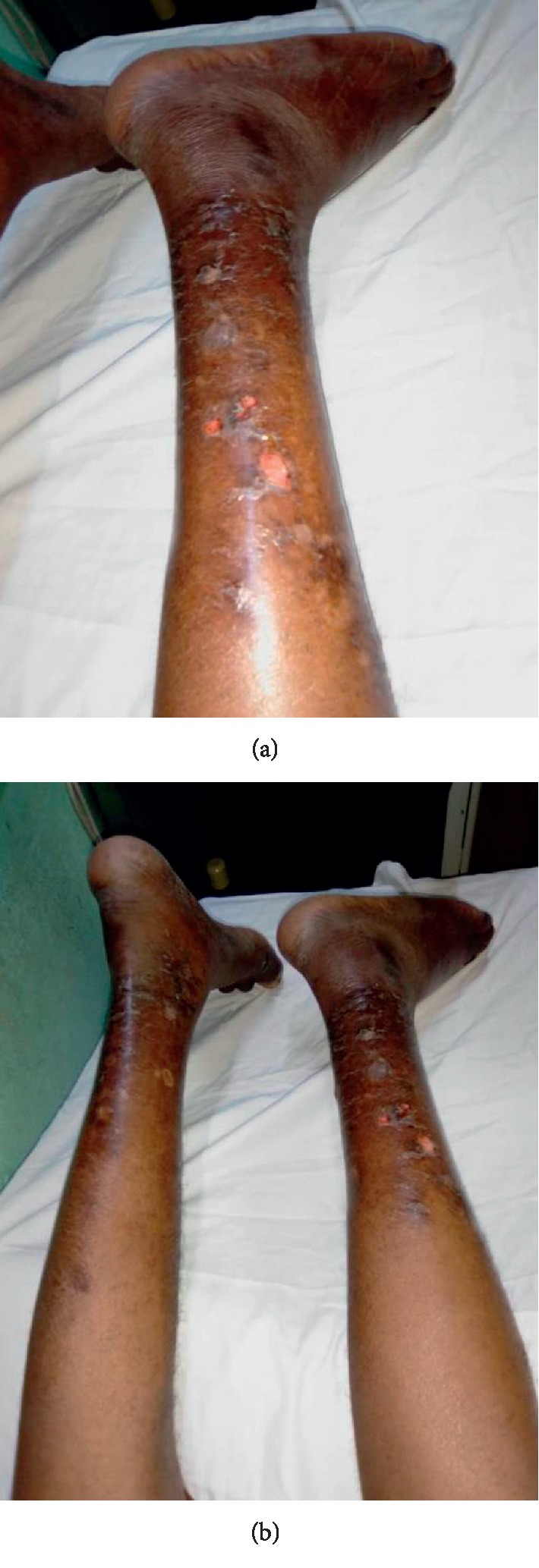
Skin xerosis complicated by postscarring ulcerations in chronic hemodialysis.

**Figure 3:**
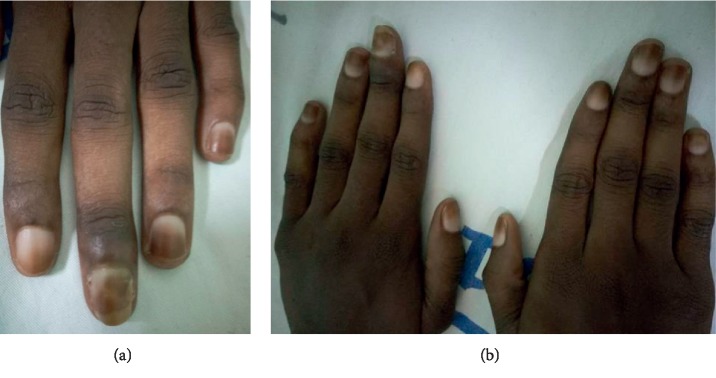
Equisegmented nails in chronic hemodialysis.

- Epidemiologically, 87 patients were included in our study with a hospital frequency of dermatological manifestations in this patient group of 33.8%. This confirms the high frequency of dermatoses in this population. The clear male predominance found is similar to the data in the literature in sub-Saharan Africa, the male sex being a factor favouring the progressive deterioration of renal function [1]. The median age of 49 years with a peak of over 50 years remains similar to the data of several authors [6–8]. This is the socially active age group, with risky behaviours such as addictive behaviours (alcohol, tobacco, drugs, and so on) and herbal medicine without discarding social stress (family and professional).
- Clinically, the most reported dermatological functional sign was pruritus (34.5%), as in most reported series [5, 6, 9]. It was generalized or localized pruritus on the back, of moderate intensity, occurring immediately after the session; Benchikhi et al. in Morocco had noted the same characteristics [10]. In our cohort, pruritus did not appear to be related to seniority in hemodialysis but rather was associated with a longer duration of hemodialysis sessions (*p* = 0.01). At the nephrology unit of the NTH-HKM in Cotonou, patients received bi-weekly dialysis for 4 hours or 5 hours or even 5.5 hours per session, whereas the European recommendations for good hemodialysis practice recommend at least three sessions per week for a minimum total of 12 hours [11]. Thus, we could suggest, among other hypotheses such as Guillet [3], that the duration of extrarenal purification, and consequently the duration of exposure to possible “allergenic factors” related to intolerance to hemodialysis equipment, could be the cause. Especially since many patients claimed a decrease in pruritus after “abundant rinsing” of the dialyzer.
- Dermatological lesions (physical skin signs) were present in 85% of cases; this result is similar to the results found in most series [9, 12–14]. Among these dermatological lesions, xerosis (Figure 2) came first in our series as in others [6, 9, 12, 15]. When it was ichthyosiform, it predominated in the legs and forearms. It was associated with a relatively high seniority. Patients over 50 years of age were most affected. Several factors are implicated in the pathogenesis of xerosis: skin ageing including glycerol deficiency with skin dryness, barrier dysfunction, chemically induced irritation, and functional abnormalities of the eccrine sweat glands [16]. Also, three out of four patients with xerosis were male. This could be explained by the fact that the sample was predominantly male, and men make less use of body cosmetics.
- The third dermatological disorder in our study was phanerial damage dominated by alopecia, nail dystrophy, and equisegmented nails (Figure 3). 14% of cases of alopecia found in our study are close to the rates of Coulibaly et al. in Burkina Faso (11.6%) and Sayeda et al. in Lebanon (12.2%) [12, 17] but far lower than the data from Brazil and Morocco which reported 33.1% and 35.8%, respectively [6, 15]. Hair dryness is thought to be due to a reduction in sebum production, and hair loss is thought to be multifactorial (stress, hyposideremia, accumulation of toxins, hypervitaminosis A, polymedication of chronic renal failure: allopurinol, cimetidine, methyldopa, iron, and beta blockers) [6, 15]. Females also influenced the occurrence of alopecia. This can be attributed to the wide variety of hair cosmetics (shampoo, oil bath, hair gel, dyes, plant extracts, fresh eggs, and so on) used by women and the traction promoted by their braids. Thus, it will be necessary to take into account these practices of women which can aggravate or accelerate alopecia related to the direct effect of hemodialysis on the hair. Nail damage was present in 22.7% of our patients. Onychopathy during chronic hemodialysis was reported both at the NTH-HKM in Cotonou and in the literature [6, 8, 13]. Rates vary from one country to another.
- Pigmentation disorders were also observed in our study; these were mainly melanoderma. It was related to seniority in hemodialysis as reported by Kouotou et al. in Cameroon [9]. This melanoderma was observed in 8% of our patients; this rate is lower than the results of other African series which report rates ranging from 17 to 94% [6, 8, 9]. It was diffuse in half of the cases in our study, sometimes in the photo-exposed areas and rarely in the palmoplantar area. This melanoderma, linked to two factors found in renal failure, can be explained by the nonelimination of pigments (urochromes and carotenoids) and anaemia, thus disrupting melanogenesis [16].
- The limitations of our study are linked on the one hand to the short duration and the small sample size and on the other hand to the unicentric nature of the study; Benin does not have many hemodialysis centres at the moment. However, this did not prevent us from achieving our main objective, which was to identify the epidemiological and clinical profile of the dermatological manifestations observed in chronic hemodialysis patients at the NTH-HKM of Cotonou (Benin).

## 5. Conclusion

Dermatological disorders in chronic hemodialysis patients at the NTH-HKM of Cotonou are common and polymorphic. They appear to be dominated by xerosis, pruritus, and phanerian disorders influenced by length per session, seniority, and gender. It is important to adapt the recommendations of good hemodialysis practice in our context and to follow-up dermatologically on this patient population.

## Figures and Tables

**Table 1:** Distribution of the 87 hemodialysis patients according to sociodemographic characteristics and antecedents (NTH-HKM, Cotonou, 2018).

	Total (*n* = 87)	Frequency (%)
Gender		
Female	29	33.3
Male	58	66.7
Age (year)		
20–30 (young)	2	2.3
31–40	19	21.8
41–50	23	26.5
>50 (elderly)	43	49.4
*Antecedents*		
Diabetes		
No	70	80.5
Yes	17	19.5
Hypertension		
No	8	9.2
Yes	79	90.8
Atopy (*n* = 87)		
No	66	75.9
Yes	21	24.1
Drug allergy (*n* = 87)		
No	62	71.3
Yes	25	28.7
Weekly frequency of sessions		
2	86	98.9
3	1	1.1
Hemodialysis seniority (Year)		
<1	19	21.8
1–2	8	9.2
3–5	26	29.9
6–10	26	29.9
11–20	7	8.0
>20	1	1.2

**Table 2:** Factors associated with the presence of alopecia in chronic hemodialysis patients at the NTH-HKM of Cotonou, Benin.

	Alopecia		*N*	*p*
	Yes	No		
	*n* (%)	*n* (%)		
Gender				
Female	9 (31.03)	20 (68.97)	29	0.004
Male	4 (6.90)	54 (93.10)	58	
Hemodialysis seniority (year)				
<1	5 (26.31)	14 (73.69)	19	0.033
1–2	2 (25.00)	6 (75.00)	8	
3–5	4 (15.38)	22 (84.62)	26	
6–10	2 (7.69)	24 (92.31)	26	
11–20	1 (14.29)	6 (85.71)	7	

**Table 3:** Factors associated with the presence of melanoderma in chronic hemodialysis patients at the NTH-HKM of Cotonou (Benin).

	Melanoderma		*N*	*p*
	Yes	No		
	*n* (%)	*n* (%)		
Hemodialysis seniority (year)				
<1	10 (52.63)	9 (47.37)	19	0.01
1–2	7 (87.50)	1 (12.50)	8	
3–5	9 (34.62)	17 (65.38)	26	
6–10	15 (57.69)	11 (42.31)	26	
11–20	1 (14.29)	6 (85.71)	7	

**Table 4:** Factors associated with the presence of xerosis in chronic hemodialysis patients at the NTH-HKM of Cotonou (Benin).

	Xerosis		*N*	*p*
	Yes	No		
	*n* (%)	*n* (%)		
Hemodialysis seniority (year)				
<1	10 (52.63)	9 (47.37)	19	0.01
1–2	7 (87.50)	1 (12.50)	8	
3–5	9 (34.62)	17 (65.38)	26	
6–10	15 (57.69)	11 (42.31)	26	
11–20	1 (14.29)	6 (85.71)	7	

**Table 5:** Factors associated with the presence of pruritus in chronic hemodialysis patients at the NTH-HKM of Cotonou (Benin).

	Pruritus		*N*	*p*
	Yes	No		
	*n* (%)	*n* (%)		
Duration per session (hour)				
4	2 (9.09)	20 (90.91)	22	0.01
4.5	6 (37.5)	10 (62.5)	16	
5	22 (44.9)	27 (55.1)	49	

## Data Availability

The epidemiological and clinical data used to support the conclusions of this study will be available to the general public after the publication of this manuscript by contacting the corresponding author.
